# An in-principle super-polynomial quantum advantage for approximating combinatorial optimization problems via computational learning theory

**DOI:** 10.1126/sciadv.adj5170

**Published:** 2024-03-15

**Authors:** Niklas Pirnay, Vincent Ulitzsch, Frederik Wilde, Jens Eisert, Jean-Pierre Seifert

**Affiliations:** ^1^ Electrical Engineering and Computer Science, Technische Universität Berlin, 10587 Berlin, Germany.; ^2^ Dahlem Center for Complex Quantum Systems, Freie Universität Berlin, 14195 Berlin, Germany.; ^3^ Fraunhofer Heinrich Hertz Institute, 10587 Berlin, Germany.; ^4^ Fraunhofer SIT, Rheinstraße 75, 64295 Darmstadt, Germany.

## Abstract

It is unclear to what extent quantum algorithms can outperform classical algorithms for problems of combinatorial optimization. In this work, by resorting to computational learning theory and cryptographic notions, we give a fully constructive proof that quantum computers feature a super-polynomial advantage over classical computers in approximating combinatorial optimization problems. Specifically, by building on seminal work by Kearns and Valiant, we provide special instances that are hard for classical computers to approximate up to polynomial factors. Simultaneously, we give a quantum algorithm that can efficiently approximate the optimal solution within a polynomial factor. The quantum advantage in this work is ultimately borrowed from Shor’s quantum algorithm for factoring. We introduce an explicit and comprehensive end-to-end construction for the advantage bearing instances. For these instances, quantum computers have, in principle, the power to approximate combinatorial optimization solutions beyond the reach of classical efficient algorithms.

## Teaser

A reduction that shows in what way one can expect quantum advantages in combinatorial optimization is presented.

## INTRODUCTION

Recent years have enjoyed an enormous interest in quantum computing as a new paradigm of computing. While ground breaking work (*1*–*3*) established that quantum computers provide a substantial speedup for certain problems over classical computers, the extent of this quantum advantage is still largely uncharted territory. It has been suggested that quantum computers may actually assist in improving existing classical algorithms for the task of combinatorial optimization. That is, the task of assigning discrete values from a finite set to finitely-many variables, such that the cost function over the variables is minimal. Here, we provide a full constructive proof that quantum computers can indeed outperform classical computers for finding approximations to combinatorial optimization problems.

**Fig. 1.**
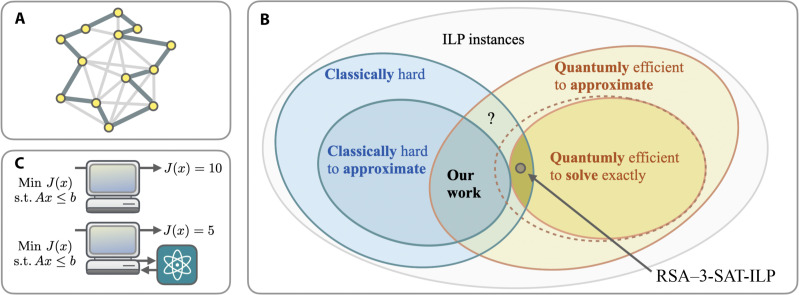
Overview of the setting of our work. (**A**) Diagrammatic sketch of the traveling salesperson problem aimed at finding the shortest possible route that visits each city (represented as vertices) exactly once and returns to the origin city. (**B**) Venn diagram that depicts the sense, in which a quantum advantage—symbolized in (**C**)—is proven in our work for integer programming problems. The gray set contains all instances of ILP, and the subsets contain the hard or respectively easy to solve instances. By hard to approximate, we mean that there is no polynomial time algorithm that approximates the size of the optimal solution up to a factor of opt^α^ · ∣*I*∣^β^, where ∣*I*∣ is the instance size; α, β are constants, such that α ≥ 0, 0 ≤ β < 1; and opt denotes the size of the optimal solution. Whether the dotted line holds true, i.e., whether there exists a problem that can be solved exactly by a polynomial-time quantum algorithm but are hard to approximate classically, is left for further research.

Combinatorial optimization problems arise in a wealth of contexts, ranging from problems in the description of nature to industrial resource optimization (*4*). In combinatorial optimization problems, one is given an objective function, which needs to be optimized over a finite set of object, such that some constraints over the objects are satisfied. A prominent example is the traveling salesperson problem, in which one has to choose a cyclic route through a set of cities, such that the length of the route is minimal (see Fig. 1A). In this case, the objective function is the sum of traveled distances along the route, which needs to be minimized. The objects are the cities and the constraints demand that the start- and endpoints are the same city, and no city is visited twice. The traveling salesperson problem underlies many routing problems that we encounter in our everyday life, such as finding the most efficient supply chain, the cheapest delivery route or the fastest three-dimensional print. In addition, job scheduling, resource allocation, or portfolio optimization—and many naturally occurring problems such as that of protein folding—can basically be seen as combinatorial optimization problems. Given the vast social and economic significance of combinatorial optimization problems, it is expected that they have been a subject of intense research for many decades. However, many problems of this kind are known to be NP-hard in worst case complexity, i.e., even the best algorithms to date cannot solve all instances of combinatorial optimization problems in tractable time. This does not mean that one cannot solve practically relevant instances up to reasonable system sizes or find good approximations to the optimal solutions. There is indeed a rich body of literature on both heuristic approaches that work well in practice (*5*) and on a rigorous theory of approximating solutions (*6*). For example, enormous traveling salesperson instances of up to 85.900 cities have been solved optimally (*7*) and there are many software suites that enable good approximations for the industry today.

Motivated by the insight that quantum computers may offer substantial computational speedups over classical computers (*1*, *2*), it has long been suggested that quantum computers may actually assist in further improving approximations to these problems. While there is no hope for an efficient either quantum or classical algorithm that is guaranteed to find the optimal solution, a crucially important question is whether quantum computers offer an advantage for combinatorial optimization problems and specifically for approximating the solution of these problems.

This topic is particularly prominently discussed in the realm of near-term quantum computers (*8*), for which full quantum error correction and fault tolerance seem out of scope but which may well offer computational advantages over classical computers (*3*, *9*). For these devices, algorithms such as the quantum approximate optimization algorithm (*10*) have been designed precisely to solve combinatorial optimization problems of the above mentioned kind. Surely, these instances of variational algorithms (*11*–*13*) will not always be able to solve these problems: At best, these algorithms may be able to produce approximate solutions that are better than those found by classical computers. They may also be able to efficiently find good approximations for more instances than classical computers when they are used perfectly. When actually operated in realistic, noisy environments, the performance of quantum devices is further reduced. For variational algorithms run on noisy devices, some obstacles have been identified for quantum computers that involve circuits that are deeper than logarithmic (*14*–*17*), obstacles that may well be read as indications that it will be challenging to achieve quantum advantages in the presence of realistic noise levels.

For variational algorithms aimed at tackling classical combinatorial optimization problems that are being cast in the form of minimizing the energy of commuting Hamiltonian terms, further obstructions are known (*18*). Some small instances of the problem can even be classically efficiently simulated [although small noise levels may help (*19*)].

The make-or-break question, therefore, is: What is, after all, the potential of quantum computers for tackling combinatorial optimization problems? A simple quantum advantage for exactly solving combinatorial optimization problems may be obtained by reducing the integer factoring problem to the 3-Satisfiability (3-SAT) problem and leveraging the advantage of Shor’s algorithm (*1*). A quantum advantage for approximating the solution of combinatorial optimization problems can also be obtained using a different proof technique than used in this manuscript. As outlined in (*20*), the celebrated PCP theorem can be used to show classical approximation hardness, while Shor’s algorithm for factoring can be used for an efficient quantum approximation algorithm. Thus, an in-principle separation between classical and quantum approximation algorithms can already be obtained from the PCP machinery and Shor’s algorithm. However, the focus of this work is to provide a technically detailed and complete proof that comprehensively describes the reductions and gives and end-to-end guidelines on how to construct the advantage-bearing combinatorial optimization instances. We expect that the concrete realization of the proof gives follow-up work additional insights over a generic proof sketch. Given the practical importance of combinatorial optimization tasks and its wide applicability, this is a valuable contribution to further advance quantum optimization algorithms.

## RESULTS

### Premise of this work

In this work, we provide a comprehensive proof that a fault-tolerant quantum computer can approximate certain combinatorial optimization problems super-polynomially more efficiently than a classical computer. While such a result can also be obtained from the PCP theorem and Shor’s algorithm (*20*), our work focuses on fully fleshing out a constructive proof to provide a clear guideline on how these advantage-bearing instances can be constructed. An important contribution of our work—particularly in the light of claims of applications of quantum computers for solving optimization problems that have become common—is also in contributing to clarifying in what precise sense one can hope for quantum advantages in optimization in the first place.

In our efforts, we digress from the PCP theorem and build on the work of Kearns and Valiant (*21*), who have shown the classical hardness of approximating the solution of the so-called formula coloring (FC) problem, a combinatorial optimization problem that generalizes the graph coloring problem. We continue to draw inspiration from (*21*) when showing an approximation hardness preserving reduction from the FC problem to integer programming [a family of combinatorial optimization problems on which variants of quantum approximation have already been applied to (*22*)]. To prove the super-polynomial quantum advantage, we extend the work of Kearns and Valiant (*21*) to show the classical approximation hardness for certain integer programming instances that are constructed from the Rivest–Shamir–Adleman (RSA) encryption function. We then provide an efficient quantum algorithm for approximating the solutions of those instances up to a polynomial factor. For a given instance *ℐ* of integer programming or FC, it can be decided in quantum polynomial time whether *ℐ* belongs to this set of advantage bearing instances.

We also formulate the hard-to-approximate instances in the optimization problem of minimizing the energy of commuting Hamiltonian terms, connecting our findings to the widely studied field of variational quantum optimization. Because the classical approximation hardness stems from the hardness of inverting the RSA encryption function (*21*), the core of the quantum advantage found in this work is ultimately essentially borrowed from, once again, Shor’s quantum algorithm (*1*) for factoring.

The kind of reasoning developed here resembles the mindset of Sweke *et al.* (*23*), Pirnay *et al.* (*24*), and Liu *et al.* (*25*) to the problem of approximating solutions to combinatorial optimization. The argument we have put forth compellingly shows that quantum computers can indeed perform provably substantially better than classical computers on instances of approximating combinatorial optimization problems, in fact, featuring a super-polynomial speedup. To make contact with quantum approximate optimization, we also spell out how the problem instances can be written in terms of Hamiltonian optimization. While the results found here are highly motivating and do show the potential of quantum devices to tackle these practically relevant problems, it remains open to which extent this potential can be unlocked for short variational quantum circuits as they are accessible in near-term quantum computers.

This result is interesting due to the technical aspects in its own right—showcasing the potential of quantum computers to offer speedups when tackling combinatorial optimization problems. It is also interesting conceptually because it provides guidance on the question what type of speedups one can expect from further quantum approximation algorithms. The present work does not suggest to solve NP-hard problems exactly on a quantum computer in polynomial time. Instead, we provide a full proof for an in-principle quantum advantage for classically hard-to-approximate combinatorial optimization problems and along the way introduce a polynomial reduction strategy. This can be seen as a positive result on the potential use of fault tolerant quantum computers and, possibly, variational quantum algorithms to address these problems.

### Technical results

Technically, in this work, we show a quantum-classical separation for the computational task of approximating combinatorial optimization problems. To show this, one needs a set of combinatorial optimization problem instances that are classically hard-to-approximate but for which we provide an efficient quantum approximation algorithm. For the classically hard-to-approximate problem instances, we build on the work of Kearns and Valiant (*21*), who have shown the classical hardness of approximating the solution of the so-called FC problem, a combinatorial optimization problem that generalizes the graph coloring problem. Before we proceed with the quantum efficiency part, we want to briefly explain the FC problem and how classical approximation hardness for specific instances can be obtained.

The FC problem is defined over a formula *F* with the integer variables *z*_1_, …, *z_m_* ∈ *ℕ*. The value of a variable acts as the color of the variable. A *k-*coloring is an assignment of colors to the *z_i_*, described by a partitioning *P* of the variable set into *k* equivalence classes, such that two variables are in the same partition if and only if they are assigned the same color. We write *z_i_* = *z_j_* if and only if the two variables are assigned the same color, and hence, they are in the same partition in *P*. Otherwise, we write *z_i_* ≠ *z_j_*. Let us now give a formal definition of the FC problem.

**Definition II.1** [FC problem (*21*)].

***Instance:*** A Boolean formula *F*(*z*_1_, …, *z_m_*), which consists of conjunctions of clauses of the form either (*z_i_* ≠ *z_j_*) or the form [(*z_i_* = *z_j_*) → (*z_k_* = *z_l_*)]*.*

***Solution:*** A minimal coloring *P* for *F*(*z*_1_, …, *z_m_*), such that *F* is satisfied.

A minimal coloring to the FC problem is a coloring with the fewest colors, i.e., ∣*P*∣ is minimal for all possible colorings, such that *F* is satisfied. To internalize, consider the example formula

$$\left(z_{1}\neq z_{2}\right)\wedge \left[\left(z_{1}=z_{3}\right)\to \left(z_{2}=z_{4}\right)\right]$$ (1)

which has the four coloring {{*z*_1_}, {*z*_2_}, {*z*_3_}, {*z*_4_}} satisfying the formula and has the minimal coloring {{*z*_1_, *z*_3_}, {*z*_2_, *z*_4_}} using only two colors while satisfying the formula. It can be easily seen that one can encode the graph coloring problem into the FC problem by constructing a formula that only consists of clauses (*z_i_* ≠ *z_j_*) for each edge in the graph between nodes *z_i_* and *z_j_*. Thus, the FC problem belongs to the computationally hard-to-solve class of NP-complete problems. In this work, we show a quantum advantage for a specific subset of FC problems that are provably hard to even approximate, but for which, we present an efficient quantum approximation algorithm. Further, we give an approximation-preserving reduction from the FC problem to the integer linear programming (ILP) problem, thus showing also a quantum advantage for integer programming.

**Definition II.2** (ILP problem).

***Instance:*** A linear objective function *J* over integer variables subject to linear constraints of the variables.

***Solution:*** A valid assignment 𝒜 of the variables under the constraints, such that the objective function *J*(𝒜) is minimal for all assignments that satisfy the constraints.

So what is this subset of classically hard-to-approximate FC/ILP problem instances? Kearns and Valiant (*21*) show how one can cleverly encode the deterministic finite automaton (DFA) that decrypts an RSA ciphertext into the FC problem. That is to say, they show how to construct a set FC problem instances FC-RSA, where if one would be able to find the smallest (or even approximately small) coloring, then one would be able to learn a DFA that could decrypt RSA ciphertexts. Because decrypting RSA ciphertexts is assumed to be intractable for classical computers, when the secret key is unknown, it follows that approximating the solutions to FC-RSA must be intractable. In this work, we substantially extend this result to ILP problems by defining a subset ILP-RSA by means of a polynomial, approximation-preserving reduction of FC-RSA to ILP. For the detailed description on how the hard-to-approximate instances are constructed and an in-depth explanation of why they are hard-to-approximate, we refer the reader to Materials and Methods. Specifically, the section “Quantum efficiency” in the Materials and Methods presents an overview of the chain of reductions and further hardness results derived in (*21*).

Building on the machinery developed in (*21*), we prove the classical approximation hardness for the combinatorial optimization task of integer programming, i.e., for the specific subset of problem instances called ILP-RSA. As described before, the instances in ILP-RSA cleverly encode the decryption of an RSA ciphertext, for which the secret cryptographic key is unknown. Hence, we obtain the following theorem, which must hold if inverting the RSA encrpytion function is computationally intractable for classical algorithms.

**Theorem II.3** (Classical hardness of approximation for linear programming). Assuming the hardness of inverting the RSA function, there exist no classical probabilistic polynomial-time algorithm that, on input, an instance ILP*_F_* of ILP-RSA finds an assignment 𝒜 of the variables in ILP*_F_*, which satisfies all constraints and approximates the optimal objective value opt_ILP_(ILP*_F_*) by

$$J\left(𝒜\right)\leq \text{op}t_{\text{ILP}}{\left(\text{IL}P_{F}\right)}^{\alpha }{|\text{IL}P_{F}|}^{\beta }$$ (2)

for any α ≥ 1 and 0 ≤ β < 1/4.

The quantity opt_ILP_(ILP*_F_*) is the minimal objective function value possible under the constraints in ILP*_F_*, and ∣ILP*_F_*∣ denotes the size of the problem instance in some fixed encoding. The theorem above essentially states that there is no classical algorithm that finds an assignment 𝒜, such that the objective value *J*(𝒜) is upper bounded by some polynomial in opt_ILP_(ILP*_F_*) times a prefactor that is determined by the size of the problem. That is under the assumption that inverting the RSA function is not possible in polynomial time on a classical computer.

However, we show that there does exist a polynomial-time quantum algorithm that finds an assignment 𝒜 of the variables that satisfies the constraints in ILP*_F_*, such that the objective value is smaller than some polynomial in opt_ILP_(ILP*_F_*).

**Theorem II.4** (Quantum efficiency for ILP-RSA). There exists a polynomial-time quantum algorithm that, on input an instance ILP*_F_* of ILP-RSA, finds a variable assignment 𝒜 that satisfies all constraints and for which the objective function is bounded as

$$J\left(𝒜\right)\leq \text{op}t_{\text{ILP}}{\left(\text{IL}P_{F_{S}}\right)}^{\alpha }$$

for all ILP*_F_* and for some α ≥ 1.

Essentially, the efficient quantum algorithm cleverly reads out the RSA parameters from an instance ILP*_F_* of ILP-RSA and then runs Shor’s algorithm for integer factorization, thereby reconstructing the secret RSA key. Given the RSA secret key, the algorithm can find an assignment of the variables in ILP*_F_*, such that the objective function is a polynomial in opt_ILP_($\text{IL}P_{F_{S}}$). This yields the sought after super-polynomial quantum advantage for approximating the optimal solution of combinatorial optimization problems. The nature of this advantage is illustrated in Fig. 1 (B and C). Note that the factor ∣ILP*_F_*∣^β^ in the hardness result (Theorem II.3) cannot decrease the approximation gap because ∣ILP*_F_*∣^β^ ≥ 1 for all β ∈ [0,1/4).

The quantum algorithm presented is distinctly not of a variational type, as they are commonly proposed for approximating combinatorial optimization tasks using a quantum computer (*10*). It is still meaningful to formulate the optimization problem as an energy minimization problem to closely connect our findings to the performance of variational quantum algorithms (*11*, *12*) in near-term quantum computing. In Materials and Methods, we give the construction on how the ILP at hand can be stated in terms of a quadratic unconstrained binary optimization problems. All these problems can be directly mapped to Hamiltonian problems where the optimal objective value is equivalent with the ground state energy of the quantum Ising Hamiltonian.

## DISCUSSION

In this work, we have made substantial progress on the important question of what potential quantum computers may offer for approximating the solution of combinatorial optimization problems. Given the social and economic impact of these problems and the large body of the recent literature on near-term quantum computing focusing on use cases of this kind, this is an important question.

We actually address this question from a fresh and unorthodox perspective. Equipped with tools from mathematical cryptography and materializing the Occam’s Razor framework in the reduction—hence settling an open question—we technically present here that we prove a super-polynomial speedup for approximating the solution of instances of NP-hard combinatorial optimization problems using a fault tolerant quantum computer. We explicitly show these speedups for instances of the much discussed ILP, which are proven to be hard to approximate by classical computations.

In this work, we provide the end-to-end construction of the advantage bearing instances, allowing further work to gain valuable insights into the quantum advantage for combinatorial optimization. Such instances are expected to prove to be useful to compare quantum versus classical optimization algorithms and provide a fruitful arena for future research in this field. The work here shows and provides guidance for the discussion of what one can reasonably hope for when discussing the potential of near-term quantum algorithms to tackle problems of combinatorial optimization.

## MATERIALS AND METHODS

### Preliminaries

#### Notation and acronyms

For what follows, some notation will be required. We will heavily build on literature from the cryptographic context and hence make use of substantial notation that is common in this context. By {0,1}*^n^*, we will denote the set of *n*-bit strings, whereas {0,1}^*^ are arbitrary finite length bit strings. 2*^X^* is the power set of *X*, for *X* being a set. 1(*a*) is the indicator function that equates to 1 if *a* is true and 0 otherwise. LSB(*x*) is the least significant bit (LSB) of *x*. ℤ*_N_* is the residue class ring *ℤ*/*N*ℤ. The application of the function binary (*x*_1_, …, *x_k_*) explicitly converts its inputs *x*_1_, …, *x_k_* to a single coherent bit string using some fixed binary encoding. The result established in this work is based on a series of reductions between various classes of computational problems and brings them together in a fresh fashion. While each of the terms is introduced explicitly in the subsequent sections, we summarize them here in Table 1 for the reader’s convenience.

**Table 1.** An overview of notation and acronyms used in this manuscript.

Acronym	Meaning	Section/definition
Eval	Evaluation problem	Definition IV.1
Con	Consistency problem	Definition IV.2
opt_Con_(*S*)	Size of the minimal consistent representation class	Learning of representations
A.P.R	approximation-preserving reduction	Reductions among representations
RSA	Rivest-Shamir-Adleman asymmetric cryptosystem	The RSA encryption function
LSB	Least significant bit	The RSA encryption function
BC	Polysize log-depth Boolean circuits	Reductions among representations
C-RSA	Boolean circuits inverting RSA	Definition IV.5
DFA	Class of deterministic finite automata	Deterministic finite automata
DFA-RSA	Subclass of DFAs computing the LSB of RSA	Classical approximation hardness for more representation classes
FC	Formula coloring problems	Definition II.1
FC-RSA	Subclass of FC encoding the solution to Con(DFA-RSA, DFA)	Approximation hardness of formula coloring
BF	Boolean formulas	Reductions among representations
BF-RSA	Subclass of BF computing the LSB of RSA	Classical approximation hardness for more representation classes
LSTM	Log-space Turing machine	Approximation hardness of formula coloring
LSTM-RSA	subclass of LSTM computing the LSB of RSA	Classical approximation hardness for more representation classes
ILP	Integer linear programs	Definition IV.10
ILP-RSA	Subclass of ILP encoding the solution to FC-RSA	Classical approximation hardness for more representation classes

#### Deterministic finite automata

DFAs (*26*) are models in computation theory, used for modeling systems with a finite number of states. A DFA is formally defined as a quintuple (*Q*, Σ, λ, *q*_0_, ω), where *Q* is a finite set of states, Σ is a finite set of symbols, constituting the automaton’s alphabet, λ : *Q* × Σ → *Q* is the transition function, *q*_0_ ∈ *Q* represents the start state, and ω ⊆ *Q* denotes the set of accept states.

**Fig. 2.**
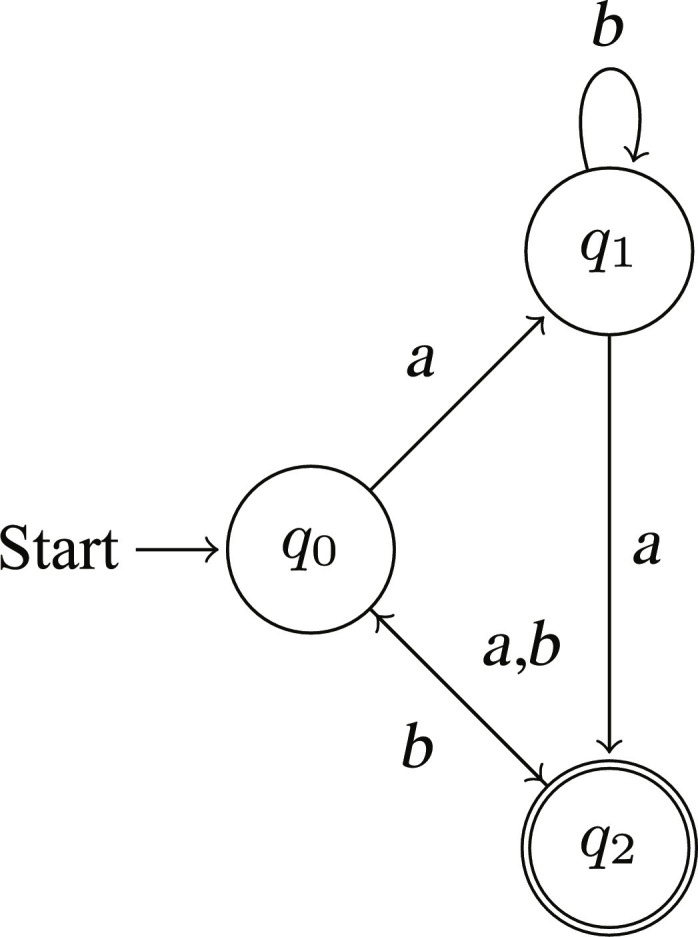
Example of a DFA. The DFA is represented as a quintuple (*Q*, Σ, λ, *q*_0_, ω), where *Q* = {*q*_0_, *q*_1_, *q*_2_}, Σ = {*a*, *b*}, λ is defined by the transitions (e.g., λ(*q*_0_, *a*) = *q*_1_, λ(*q*_0_, *b*) = *q*_2_, etc.), *q*_0_ is the initial state, and ω = {*q*_2_} is the set of accept states.

The DFA operates on a string composed of symbols from Σ. Beginning from the start state *q*_0_, it transitions between states according to the transition function λ. Upon processing the entire string, if the DFA is in a state that is part of ω, then the string is accepted by the DFA; otherwise, it is rejected. Figure 2 presents a graphical illustration of an exemplary DFA. It is known that DFAs recognize exactly the set of regular languages (*26*).

#### Representation classes

To show a quantum-classical separation for a computational task, one needs a classically hard problem. Many computational tasks that are hard for classical computers may be derived from cryptography, where it can be shown that under cryptographic *assumptions* (such as “factoring is hard”), learning certain concepts or properties about a specific cryptographic function is hard. In particular, in this work, we are concerned with how these concepts are represented and how large these representations are. To do this, let us introduce the notion of representation classes that capture the model of concepts in a precise manner. Let *X* ⊆ {0,1}^*^ be a set of binary strings with finite length, called the domain, which encodes all objects of interest to us. For example, *X* may be the set of all images or *X* may be the set of all music songs. A concept over *X* is described by a subset of *X*, which is defined via {*x* ∈ *X* ∣ concept is true for *x*}. A concept may be for example “depicts a tree” or “is a happy song.”

**Fig. 3.**
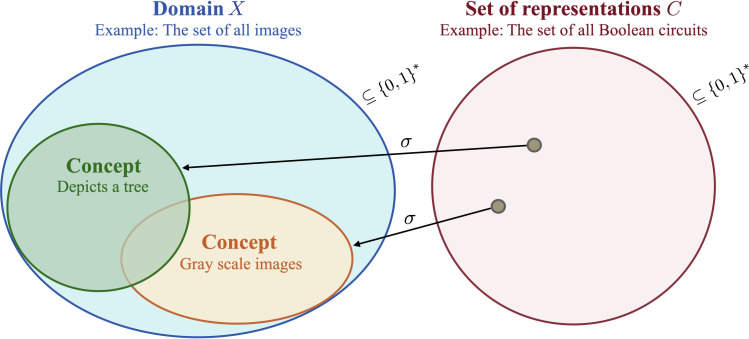
The interplay between representations and concept. The domain *X* can formally be seen as a set of finite bit strings. Concepts are subsets of the domain, which can be described by representations *c* ∈ *C*. Together with the map σ, mapping representations to concepts, the tuple (σ, *C*) is called a representation class.

We are in particular interested in how a concept is represented. Different representations for a concept can, for example, be Boolean circuits, Boolean formulae, Turing machines, or DFA (*26*). We, therefore, define a representation class over *X* to be the pair (σ, *C*), where *C* ⊆ {0,1}^*^ is the set of representation descriptions, for example, the set of descriptions for Boolean circuits or finite automata. The function σ : *C* → 2*^X^* maps a representation description to a concept. For example, σ maps a DFA to the set of bit strings that it accepts or a Boolean formula to its satisfying assignments. We will sometimes denote (σ, *C*) simply by *C* if σ is clear from the context. Figure 3 visualizes the relationship between representations and concepts.

Observe that for all *c* ∈ *C*, σ(*c*) is a concept over *X* and the entire image space σ(*C*) is called the concept class represented by the representation class (σ, *C*). We denote by ∣*c*∣ the length of the representation description using some standard encoding. In addition, for a representation *c* ∈ *C*, we denote by *c*(*x*) = 1 [if *x* ∈ σ(*c*)] the label of *x* under the concept σ(*c*), with the index function 1. Furthermore, a labeled sample

$$S=\left\{\left[x,c\left(x\right)\right]|x\in \tilde{X}\subseteq X\right\}$$ (3)

of a concept σ(*c*) is a set of labeled examples from a subset $\tilde{X}$ of the domain *X*. Note that a sample consists of multiple examples. Last, let (ϕ, *H*) be another representation class over *X* and let *D* be a probability distribution over *X*. For any *h* ∈ *H*, we define the error of *h* under *D* with respect to a target representation *c* as

$$\text{erro}r_{c,D}\left(h\right)=Pr_{x∼D}\left[c\left(x\right)\neq h\left(x\right)\right]$$ (4)

#### Polynomial-time reductions

Polynomial-time reductions are an important building block of this work, as they will be an integral part of our proof of a quantum-classical computational separation for combinatorial optimization problems. Building on the work of Kearns and Vazirani (*27*), reductions are required to “carry over” classical hardness results of representation learning to combinatorial optimization problems. At the same time, we find that quantum computers can break the construction and lead to a quantum advantage for combinatorial optimization. Let us now introduce the notion of general polynomial-time reductions among computational problems.

Let *A*, *B* be two computational problems. Consider the function τ to map an instance 𝒜 of *A* to an instance τ(𝒜) of *B*. Furthermore, let *g* be a function that maps from the solution space of *B* to the solution space of *A*. The pair of functions (τ, *g*) is a polynomial-time reduction from *A* to *B*, if τ, *g* are computable in polynomial time and if *y_ℬ_* is a solution of τ(𝒜) if and only if *g*(*y_ℬ_*) is a solution of *A*.

Note that while τ maps instances from *A* to *B*, *g* works in the backward direction, mapping solutions of *B* to *A*. This will be important for reductions between combinatorial optimization problems. In some cases, where *g* is the identity, we call the reduction simply by the instance transformation τ. Further, we denote by *A*≤*_p_B* (“*A* polynomial time reduces to *B*”) if there exists a polynomial-time reduction from *A* to *B*. Note that because the run time of τ and *g* are at most polynomial in their inputs, the outputs can be larger than the inputs at most by a factor of poly(∣𝒜∣), poly(∣*y_ℬ_*∣), respectively.

#### Reductions among representations

To understand our proof of the quantum advantage in combinatorial optimization, we require polynomial-time reductions among the evaluation problem of representation classes. Intuitively, these reductions show that one representation class is at least as powerful as another and that they can be transformed into each other. In (*21*), these reductions have been used to derive (classical) computationally hard problems for different representations. First we define a technical construction, the evaluation problem.

**Definition IV.1** [Evaluation problem Eval(*C*)]*.*

***Instance:*** The pair (*c*, *x*), where *C* is a representation class over the domain *X*, *c* ∈ *C* is a representation description, and *x* ∈ *X.*

***Solution:*** The result *c*(*x*) of *c* on *x.*

Let *n* ∈ *ℕ* and define BC*_n_* to be the representation class of polynomially evaluable Boolean circuits with domain *X* = {0,1}*^n^* and with depth *O*[log (*n*)] and size *O*[poly(*n*)], and let BC = ∪_*n*≥1_BC*_n_*. In a similar manner, define BF to be the representation class of Boolean formulae of polysize, define LSTM to be the representation class of log-space Turing machines and, lastly, define DFA to be the representation class of deterministic finite automata (*26*) of polysize. It holds that

$$\text{Eval}\left(\text{BC}\right)\leq_{p}\text{Eval}\left(\text{BF}\right)$$ (5)

$$\text{Eval}\left(\text{BF}\right)\leq_{p}\text{Eval}\left(\text{LSTM}\right)$$ (6)

$$\text{Eval}\left(\text{LSTM}\right)\leq_{p}\text{Eval}\left(\text{DFA}\right)$$ (7)

Subsequently, we sketch the proof ideas for the three reductions above. The full proofs can be found in (*27*) and (*21*). From here on after, we consider *n* to be the size of the input to a Boolean circuit in BC.

In Eq. 5 We denote this polynomial-time reduction by τ_1_. Recall that τ_1_ is the instance transformation algorithm and the solution transformation is the identity. Let *c* be a Boolean circuit in BC with depth *d* = *O*[log (*n*)] and size *s* = *O*[poly(*n*)]. Every Boolean circuit can be identified with a directed acyclic graph where each vertex has fan-in at most 2. The instance transformation in the reduction goes by starting at the output vertex of *c* and recursively building the Boolean formula *f* by walking back through *c* and substituting clauses in *f*. *f* will then consist of at most 2*^d^* clauses over *n* variables, which is size *O*[poly(*n*)]. Clearly, *c* and *f* compute the same function, the reduction (Eq. 5) holds because the transformation can be performed by an *O*[poly(*n*)]-time algorithm. We have τ_1_(*c*, *x*) = (*f*, *x*).

In Eq. 6 we denote this polynomial-time reduction by τ_2_. This reduction uses the fact that we can transform any Boolean formula *f* to a log-space Turing machine *m* that, on input, *x* computes *m*(*x*) = *f*(*x*) in time *O*[poly(*n*)]. The details for this transformation can be found in (*27*). Again, we denote the operation of this instance transformation algorithm as τ_2_(*f*, *x*) = (*m*, *x*).

In Eq. 7 we denote this polynomial-time reduction by τ_3_. The reduction uses a transformation of log-space Turing machines to DFA (*26*). In particular, for each log-space Turing machine *m*, one can construct a DFA *t* that, on input of polynomially, many copies of the original input *x* simulates *m* (*27*). Note that in the reduction here, the input is transformed, such that *x* is repeated *p*(*n*) many times and then taken as the input to *t*, where *p* is a polynomial in *n*. We thus have $\tau_{3}\left(m,x\right)=\left[t,\underset{p\left(n\right)\text{times}}{\underbrace{x,\ldots ,x}}\right]$ , such that

m(x)=t(x,…,x) (8)

Sometimes we are only interested in the transformation of the representation description and not in the input *x*. If we say that we transform a representation description *c* using τ_1,2,3_, then we omit the second input *x* and simply write τ_1,2,3_(*c*). Recall that in the reductions above, because the instances are transformed by polynomial-time algorithms, the output instances can be larger than the input at most by a polynomial factor.

#### Learning of representations

To obtain a classical hardness result for approximation tasks, the work of Kearns and Valiant (*21*) use the so-called Occam learning framework (*28*). Generally speaking, the Occam learning framework makes a connection between nearly minimal hypotheses, which are consistent with observations and the ability to generalize from the observed data in the sense of probably approximately correct (PAC) learning. To introduce this formalism, let (σ, *C*), (ϕ, *H*) be two representation classes over the domain *X* ⊆ {0,1}*^n^*. In the following, we write *C* for (σ, *C*) and *H* for (ϕ, *H*) and denote the two representation descriptions *c* ∈ *C* and *h* ∈ *H* as elements of the set of representation descriptions of (σ, *C*) and (ϕ, *H*). Given a labeled sample

$$S=\{[x_{1},c(x_{1})],\ldots ,[x_{m},c(x_{m})]\}$$ (9)

of *m* examples, we say that *h* ∈ *H* is consistent with *S* if and only if *c*(*x_i_*) = *h*(*x_i_*) for all *i* = 1, …, *m*. The *x*_1_, …, *x_m_* ∈ *X* might be drawn at random according to a distribution *D* over *X*. We denote by opt_Con_(*S*) the size of the smallest *h* ∈ *H* that is consistent with *S*. The consistency problem is defined as follows:

**Definition IV.2** [Consistency problem Con(C, H) (*21*)]*.*

***Instance:*** A labeled sample *S* of some *c* ∈ *C.*

***Solution:*** *h* ∈ *H*, such that *h* is consistent with *S* and ∣*h*∣ is minimized.

We denote by Con(*C*, *H*) the problem of finding a minimal *h* ∈ *H* that is consistent with some labeled sample *S* of some *c* ∈ *C*, and likewise, we call such a minimal consistent *h* a solution to the consistency problem of an instance *S* of Con(*C*, *H*). Occam’s razor makes a connection between the consistency problem and the ability to learn one representation class by another. In this context, learning is defined as follows: Let 0 ≤ ϵ < 1 and 0 < δ ≤ 1. An (ϵ, δ)-PAC (*29*) learning algorithm for *C* by *H* outputs an *h* ∈ *H*, such that error_*c*,*D*_(*h*) ≤ ϵ with a probability at least 1 − δ (for all distributions *D* over *X* and all *c* ∈ *C*).

We are now in the position to introduce the core theorem of this section, which connects the task of PAC learning and an approximation task. Intuitively, the following theorem states that finding a hypothesis that explains the observed data (i.e., is consistent with *S*) and is substantially more compact than the data is sufficient for PAC learning.

**Theorem IV.3** [Occam’s razor (*21*, *28*)]. Given a labeled sample *S* of *c* of size

$$m=O\left[\frac{1}{\epsilon }\text{log}\frac{1}{\delta }+{\left(\frac{n^{\alpha }}{\epsilon }\text{log}\frac{n^{\alpha }}{\epsilon }\right)}^{1/\left(1-\beta \right)}\right]$$ (10)

where the *m* examples have been sampled independently from *D* and for some fixed α ≥ 1 and 0 ≤ β < 1, any *h* that is consistent with *S* and which satisfies

$$|h|\leq \text{op}t_{\text{Con}}{\left(S\right)}^{\alpha }{|S|}^{\beta }$$ (11)

does also satisfy error_*c*,*D*_(*h*) ≤ ϵ with a probability at least 1 − δ.

Here, α and β are fixed values for the Occam’s razor prescription, the intuition for them being hinted at in (*28*). When *m* is fixed to a sufficiently large number, fulfilling the scaling of the above theorem, then α can be seen as reflecting the property that opt_Con_(*S*)^α^ bounds some polynomial in opt_Con_(*S*) and β can hence be viewed as a “compression parameter.” If β = 0, we have complete compression. Then, the algorithm provides a consistent hypothesis of complexity at most opt_Con_(*S*)^α^, independent of the sample size. The sample size needed is then $m=O\left(\frac{1}{\epsilon }\text{log}\frac{1}{\delta }\right)$ . For β → 1, we actually have not learned much because almost all of *S* can be encoded in *h*.

Then, note that the size of *S* is a polynomial in $\left(n,\frac{1}{\epsilon },\frac{1}{\delta }\right)$ . The variable α resembles that ∣*h*∣ must be smaller than some polynomial in the optimal solution size, while β forces that *h* does not simply hard-encode *S*. Clearly, it follows that any algorithm that for all *c* ∈ *C* and all *D* on input *S* sampled according to *D* of size $\text{poly}\left(n,\frac{1}{\epsilon },\frac{1}{\delta }\right)$ outputs an *h* ∈ *H* with ∣*h*∣ upper bounded as in the “Theorem IV.3” is a PAC learning algorithm for *C* by *H*. Learning *C* by *H* can be interpreted as an approximation task. Specifically, the task is to approximate the optimal solution opt_Con_(*S*), which is the size of the smallest representation consistent with *S* by ∣*h*∣, where *h* is a representation that is also consistent with *S* for any *S* of sufficient size. An algorithm achieving such an approximation within a factor of opt_Con_(*S*)^α−1^∣*S*∣^β^, for all *S* with $|S|=\text{poly}\left(n,\frac{1}{\epsilon },\frac{1}{\delta }\right)$ , is an (ϵ, δ)-PAC learner for *C*. In the remainder of this work, when we say that some “algorithm approximates the solution of the Con(*C*, *H*) problem,” we mean that the algorithm outputs an *h*, such that ∣*h*∣ approximates opt_Con_(*S*) by a factor opt_Con_(*S*)^α−1^∣*S*∣^β^, where *h* has the important property of being consistent with *S*. This sense of approximation might seem unnatural, but the Con problem will later be reduced to a combinatorial optimization task, where it is natural to approximate some scalar quantity and satisfy some constraints.

#### *Formula* coloring *problem*

We now introduce the formula coloring problem (FC) that takes the center stage in our later argument. It is a combinatorial optimization problem that has originally been introduced in (*21*) as a generalization of the more common graph coloring problem. It is an optimization problem of the type as is frequently considered in notions of quantum approximate optimization: In a subsequent section, we will formulate this problem as a problem of minimizing the energy of a commuting local Hamiltonian to make that connection explicit. It is one of the main results of this work to show a super-polynomial quantum advantage for FC and integer programming. Let *z*_1_, …, *z_m_* ∈ *ℕ* be the variables in a Boolean formula, each being assigned an integer value, which acts as the integer valued color of the variable. That is to say, each of the variables *z*_1_, …, *z_m_* takes exactly one of the possible values referred to as colors. We regard an assignment of colors to the *z_i_* (called a coloring) as a partition of the variable set into equivalence classes. That is to say, two variables have the same color if and only if they are in the same equivalence class. For the FC problem, we consider Boolean formulae *F*(*z*_1_, …, *z_m_*), which consist of conjunctions of two types of clauses. On the one hand, these are clauses of the form (*z_i_* ≠ *z_j_*). This is, in fact, precisely of the form as the clauses of the more common graph coloring problem. On the other hand, there are clauses of the form [(*z_i_* = *z_j_*) → (*z_k_* = *z_l_*)]. This material conditional, as it is called in Boolean logic, can equivalently and possibly more commonly be written as

$$\left[\left(z_{i}\neq z_{j}\right)\vee \left(z_{k}=z_{l}\right)\right]$$ (12)

A coloring is an assignment of colors to the *z_i_*, described by a partitioning *P* of the variable set into *k* equivalence classes, i.e., ∣*P*∣ = *k*. This means that *z_i_* = *z_j_* if and only if they are in the same partition of the *k* partitions in *P*. We are now in the position to formulate the FC problem.

**Definition IV.4** [Formula coloring problem FC (*21*)].

**Instance:** A Boolean formula *F*(*z*_1_, …, *z_m_*) which consists of conjunctions of clauses of the form either (*z_i_* ≠ *z_j_*) or the form [(*z_i_* = *z_j_*) → (*z_k_* = *z_l_*)].

**Solution:** A minimal coloring *P* for *F*(*z*_1_, …, *z_m_*) such that *F* is satisfied.

A *minimum solution* to the FC problem is a coloring with the fewest colors, i.e., ∣*P*∣ is minimal for all possible colorings, such that *F* is satisfied. The example given in (*21*) is the formula

$$\left(z_{1}=z_{2}\right)\vee \left[\left(z_{1}\neq z_{2}\right)\wedge \left(z_{3}\neq z_{4}\right)\right]$$ (13)

has as a model the two-color partition {*z*_1_, *z*_3_}, {*z*_2_, *z*_4_} and has as a minimum model the one-color partition {*z*_1_, *z*_2_, *z*_3_, *z*_4_}. The FC problem is obviously NP-complete, as the problem is in NP and graph coloring is NP-hard.

#### The RSA encryption function

Throughout this work, we will make use on the hardness of inverting the RSA encryption function (*30*), which forms the foundation of the security of the RSA public key cryptosystem, one of the canonical public key crypto systems and presumed to be secure against classical adversaries (*31*).

Let *N* = *p* × *q* be the product of two primes *p* and *q*, both of similar bit length. Define Euler’s totient function ϕ, where ϕ(*N*) is equal to the number of positive integers up to *N* that are relative prime to *N*. It holds that *x*^ϕ(*N*)^ = 1 mod *N*. When two parties, which we refer to as Bob and Alice, wish to communicate via an authenticated but public channel, they can do so as follows: First, Alice generates two primes *p* and *q* of similar bit length and computes their product *N* = *p* × *q*. Then, Alice generates a so-called public-private key pair (*d*, *e*), where *d* is the secret key satisfying *d* × *e*modϕ(*N*) = 1 and *e* is the public exponent. Alice shares the public key (*e*, *N*) with Bob over the public channel. We define the RSA encryption function for a given exponent *e*, a message *x* ∈ ℤ*_N_*, and a modulus *N* as

$$\text{RSA}\left(x,N,e\right)=x^{e}\text{mod}\;N$$ (14)

To encrypt a message *x* ∈ *ℤ_N_*, Bob simply computes the output of the RSA encryption function, given *N* and *e*. Bob then sends the ciphertext *c* = *x^e^* mod *N* to Alice, who decrypts the ciphertext by computing *c^d^* mod *N* = (*x^e^*)*^d^* mod *N* = *x*^1+*i*×ϕ(*N*)^ mod *N* = *x* mod *N*, where the last step follows from the fact that *x*^ϕ(*N*)^ = 1 mod *N* and *e* × *d* = 1 + *i* × ϕ(*N*) for some *i* ∈ *ℕ* because *e* × *d* mod ϕ(*N*) = 1.

The security of the RSA cryptosystem is closely related on the presumed hardness of integer factoring and, more generally, is based on the presumed hardness of inverting the RSA encryption function without knowledge of the secret key *d*. That is, there is no known classical polynomial-time algorithm that, given [RSA(*x*, *N*, *e*), *N*, *e*] outputs *x*. On a quantum computer, however, Shor’s algorithm (*1*) can be used to factor the integer *N* in polynomial time. This immediately gives rise to a quantum polynomial time algorithm that inverts the RSA encryption function—simply factor the public modulus using Shor’s algorithm and then compute ϕ(*N*) = (*p* − 1) × (*q* − 1). Then, one can find a *d*, such that *e* × *d*modϕ(*N*) = 1 by using the extended Euclidean algorithm. In summary, under the standard cryptographic assumption that the RSA encryption function is hard to invert, Shor’s algorithm thus gives rise to a computational quantum-classical separation. As we will show, this separation extends to the approximation of combinatorial optimization problems as well.

Throughout this work, we will make use of the fact that determining the LSB of *x*, given that RSA(*x*, *N*, *e*) is as hard as inverting the RSA encryption function. Formally, Alexi *et al.* (*32*) have proven that if there exists a classical polynomial-time algorithm that finds the LSB of *x*, given *RSA*(*x*, *N*, *e*), then there exists a classical polynomial-time algorithm that inverts the RSA encryption function.

### Classical hardness of approximation

**Fig. 4.**
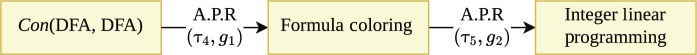
The reduction chain from the consistency problem to combinatorial optimization problems. In "Approximation hardness of the Con problem," we introduce Boolean circuits, whose sizes are hard to approximate by ∣*h*∣, where *h* is a hypothesis that is consistent with a sample labeled by the circuits. This directly implies the approximation hardness of Con(DFA, DFA). In "Approximation hardness of formula coloring," we present an approximation-preserving reduction from Con(DFA, DFA) to FC (*21*). We then extend the results of Ref. (*21*) by showing in "Approximation hardness of integer linear programming" an approximation-preserving reduction from FC to ILP, yielding the approximation hardness for ILP.

To show our quantum advantage, we require a classical hardness result and quantum efficiency result. In this section, we establish the classical hardness of approximating combinatorial optimization solutions. We build on the results of Kearns and Valiant (*21*), where the hardness of approximation tasks has been established. Furthermore, their work shows how these hard-to-approximate problems can be reduced to the combinatorial optimization problem of FC. We then extend these results by showing an approximation-preserving reduction from FC to ILP. These results will constitute the classical hardness part for the quantum-classical separation we show. Figure 4 gives a high level overview of the results presented in this section.

#### Approximation hardness of the Con problem

In this subsection, we present the result that approximating the solution of Con(DFA, DFA) is hard using a classical computer (*21*). This result is obtained through the assumption that inverting the RSA encryption function is hard, a widely accepted cryptographic assumption. To do this, one defines a class of Boolean circuits that essentially decrypt a given RSA ciphertext and output the LSB of the clear text. Intuitively, the authors in (*21*) show that, because PAC learning these Boolean circuits is hard (otherwise one would be able to invert RSA), the approximation of these decryption circuits by any polynomially evaluable representation class in the sense of the “Theorem IV.3” must also be hard using a classical computer. They then show that this implies that approximating the solution of Con(DFA, DFA) must also be hard. To follow the argumentation in (*21*), let *N* ∈ *ℕ* and *x* ∈ *ℤ_N_* and define

$$\begin{array}{c} \text{power}s_{N}\left(x\right)≔x\text{mod}N,x^{2}\text{mod}N,x^{4}\text{mod}N,\ldots \\ \ldots ,x^{2^{\text{log}\left(N\right)}}\text{mod}N \end{array}$$ (15)

as the sequence of the first log(*N*) + 1 square powers of *x*.

**Definition IV.5** [Boolean circuit for the LSB of RSA (*21*)]. Let C-RSA*_n_* ⊂ *BC_n_* and C-RSA = ⋃_*n*≥1_ C-RSA*_n_* be the representation class of log-depth, poly-size Boolean circuits that on input binary{powers*_N_*[RSA(*x*, *N*, *e*)], *N*, *e*} and output LSB(*x*) for all *x* ∈ *ℤ_N_.* Each representation in C-RSA*_n_* is defined by a triple (*p*, *q*, *e*), and this representation will be denoted as *r*_(*p*,*q*,*e*)_, where *p* and *q* are primes of exactly *n*/2 bits and *e* ∈ *ℤ_N_* and *N* = *p* · *q.*

An example of *r*_(*p*,*q*,*e*)_ ∈ C-RSA*_n_* is of the form

$$\left(\text{binary}\{\text{power}s_{N}[\text{RSA}(x,N,e)],N,e\},\text{LSB}(x)\right)$$ (16)

with *x* ∈ *ℤ_N_*.

**Fig. 5.**
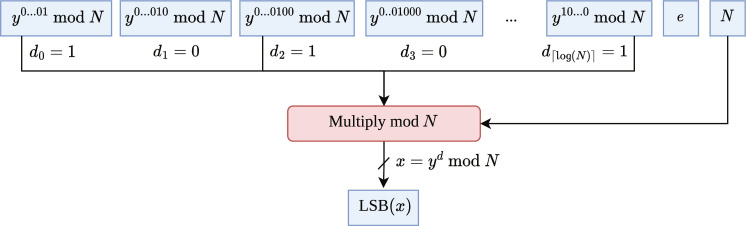
A Boolean circuit in the class C-RSA. The input to the circuit in C-RSA is the power sequence of the RSA ciphertext of RSA(*x*, *N*, *e*) = *y*. The circuit computes the LSB of *x* by simply performing modular multiplication on the 2*^i^*th powers of the power sequence where the secret key bit *d_i_* = 1, for the secret key *d*. Thereby, the secret key *d* is hard-wired into the circuit, and the decryption *x* = *y^d^* mod *N* is explicitly performed. This can be done in an *O*[log (*n*)] deep circuit (*36*).

It is important to note at this point that the calculation of the LSB of *x*, given the input binary{powers*_N_*[RSA(*x*, *N*, *e*)], *N*, *e*} can indeed be performed by a *O*[log (*n*)]-depth, *poly*(*n*)-size Boolean circuit if the decryption key *d* is known (*21*). In Fig. 5, we depict a schematic picture of such a Boolean circuit in C-RSA. Because learning the LSB of the cleartext is as hard as inverting the RSA function (*32*), which is widely assumed to be intractable for classical computers, Kearns and Valiant (*21*) show the classical approximation hardness of Con(C-RSA, *H*), where *H* is any polynomially evaluable representation class. The following theorem states that (assuming the classical hardness of inverting RSA) and given some sample *S* of some *r*_(*p*,*q*,*e*)_ ∈ C-RSA, no polynomial-time classical algorithm can output a hypothesis *h* ∈ *H* that is consistent with *S* and only polynomially larger than the smallest possible hypothesis.

**Theorem IV.6** [Classical approximation hardness of C-RSA (*21*)]. Let *H* be any polynomially evaluable representation class. Assuming the hardness of inverting the RSA function, there exists no classical probabilistic polynomial-time algorithm that on input an instance S of Con(C-RSA, *H*) finds a solution *h* ∈ *H* that is consistent with *S* and approximates the size opt_Con_(*S*) of the optimal solution by

$$|h|\leq {\left[\text{op}t_{\text{Con}}\left(S\right)\right]}^{\alpha }{|S|}^{\beta }$$

for all *S* and any α ≥ 1 and 0 ≤ β < 1.

Because $|S|=n\times m=n\times \text{poly}\left(n,\frac{1}{\epsilon },\frac{1}{\delta }\right)$ and α ≥ 1 we get that the optimal size opt_Con_(*S*) cannot be approximated up to a polynomial factor, holding for all classical probabilistic polynomial-time algorithms, where the sense of approximation is explained in detail in “Learning of representations.”

#### Classical approximation hardness for more representation classes

Furthermore, Kearns and Valiant (*21*) show that the approximation hardness of C-RSA implies approximation hardness for Boolean formulae, log-space Turing machines, and DFAs. In particular, let BF-RSA be the class of Boolean formulae that we obtain when we reduce every instance in C-RSA using τ_1_, i.e., BF-RSA = {*F*∣(*F*, *x*) = τ_1_(*I*)andIinstanceofEval(C-RSA)}. In a similar manner, LSTM-RSA is the class of log-space Turing machines that we obtain when we reduce B-RSA using τ_2_, and lastly, DFA-RSA is the class of DFAs that we obtain when using τ_3_ on BF-RSA. Because the evaluation problem of resulting representations are poly-time reducible to each other and are at most polynomially larger, the following holds (*21*):

**Theorem IV.7** [Classical approximation hardness of more representations (*21*)]. Let *H* be any polynomially evaluable representation class. Assuming the hardness of inverting the RSA function, there exists no classical probabilistic polynomial-time algorithm that, on input an instance *S* of (i) Con(BF-RSA, *H*), (ii) Con(LSTM-RSA, *H*)*,* or (iii) Con(DFA-RSA, *H*), finds a solution *h* ∈ *H* that is consistent with *S* and approximates the size opt_Con_(*S*) of the optimal solution by

$$|h|\leq {\left[\text{op}t_{\text{Con}}\left(S\right)\right]}^{\alpha }{|S|}^{\beta }$$

for all *S* and any α ≥ 1 and 0 ≤ β < 1. Specifically, note that approximating the solution of Con(DFA-RSA, DFA) is at least as hard as to approximate the solution of Con(DFA-RSA, *H*).

#### Approximation hardness of formula coloring

In this work, we are interested in showing a quantum advantage for approximating the solution of combinatorial optimization problems. Therefore, we require a classical approximation hardness result for a combinatorial optimization problem. To that end, the work of Kearns and Valiant (*21*) gives an approximation-preserving reduction from the Con(DFA, DFA) problem to the FC problem, which is a combinatorial optimization problem. We denote the approximation-preserving reduction from Con(DFA, DFA) to FC by (τ_4_, *g*_1_), where we will explicitly give the construction of the instance transformation τ_4_, which maps an instance *S* of Con(DFA, DFA) to an instance *F_S_* of FC. First, observe that *S* contains the examples (*w*_1_, *b*_1_), (*w*_2_, *b*_2_), …, (*w_m_*, *b_m_*), where *w_i_* ∈ {0,1}*^k^* and the labels *b_i_* ∈ {0,1}. The formula *F_S_* will be over variables $z_{i}^{j}$ , where 1 ≤ *i* ≤ *m* and 0 ≤ *j* < *k*. Essentially, each variable $z_{i}^{j}$ will correspond to the state that a consistent DFA would be in after reading the *j*th bit of *w_i_*.

We now give the construction for the formula *F_S_*: For each *i*_1_, *i*_2_ and *j*_1_, *j*_2_, such that 0 ≤ *j*_1_, *j*_2_ < *k* and $w_{i_{1}}^{j_{1}+1}=w_{i_{2}}^{j_{2}+1}$ , we add the predicate

$$\left[\left(z_{i_{1}}^{j_{1}}=z_{i_{2}}^{j_{2}}\right)\to \left(z_{i_{1}}^{j_{1}+1}=z_{i_{2}}^{j_{2}+1}\right)\right]$$ (17)

to the conjunctions in *F_S_*. Intuitively, this encodes that for two inputs, *w*_*i*_1__, *w*_*i*_2__, a DFA that is in the same state for both inputs and then reads the same symbol for both those strings next, the resulting state should also be the same. To ensure that the DFA is consistent with the labels of the sample as well, for each 1 ≤ *i*_1_, *i*_2_ ≤ *m*, such that *b*_*i*_1__ ≠ *b*_*i*_2__, we add the predicate

$$\left(z_{i_{1}}^{k}\neq z_{i_{2}}^{k}\right)$$ (18)

to the conjunctions in *F_S_*. Those clauses encode the fact that for different labels, the states (after reading the whole input) of a consistent DFA must be different because any state can either only accept or reject.

If ∣*S*∣ is the number of bits in *S*, then the resulting *F_S_* consists of Θ(∣*S*∣ 2) many clauses. For the solution transformation *g*_1_, as well as the proof that this reduction is indeed correct, we refer to the proof in (*21*). Note that by the construction above, the bits of the examples are now encoded in the clauses of *F_S_* together with the correct working of the DFA and the solution (the structure of the minimal DFA) is the minimal coloring of *F_S_*. Because of the results in (*21*), the following theorem holds:

**Theorem IV.8** [Reduction of Con(DFA, DFA) to FC (*21*)]. There is a polynomial time algorithm τ_4_ that on input an instance S of the problem Con(DFA, DFA) outputs an instance *F_S_* of the FC, problem such that *S* has a *k*-state consistent hypothesis *M* ∈ DFA if and only if *F_S_* has a coloring *P*, with ∣*P*∣ = *k.*

Note that the algorithm τ_4_ is precisely the instance transformation of the reduction (τ_4_, *g*_1_), and we have

$$\text{Con}\left(\text{DFA},\text{DFA}\right)\leq_{p}\text{FC}$$ (19)

In particular, it holds that

$$\text{Con}\left(\text{DFA-RSA},\text{DFA}\right)\leq_{p}\text{FC-DSA}$$ (20)

where FC-RSA is the class of FC problems that result out of running τ_4_ (introduced in “Approximation hardness of formula coloring”) on the instances in the problem Con(DFA-RSA, DFA). In particular, *g*_1_ transforms the minimal solution of FC into the minimal solution of Con(DFA, DFA), thus opt_FC_(*F_S_*) = opt_Con_(*S*) (due to Theorem IV.8) and ∣*F_S_*∣ = Θ(∣*S*∣ 2). From those two facts, it follows that finding a valid coloring *P* of *F_S_*, such that ∣*P*∣ ≤ opt_FC_(*F*)^α^∣*F*∣^β′^, would contradict Theorem IV.7 for the parameter range α ≥ 1*,* 0 ≤ β′ < 1/2. Thus, the reduction (τ_4_, *g*_1_) preserves the approximation hardness of Con(DFA-RSA, DFA) in the sense of the following theorem (*21*):

**Theorem IV.9** [Classical hardness of approximation for coloring (*21*)]. Assuming the hardness of inverting the RSA function, there exists no classical probabilistic polynomial-time algorithm that on input an instance *F_S_* of FC-RSA finds a valid coloring *P* that approximates the size opt_FC_(*F_S_*) of the optimal solution by

$$|P|\leq \text{op}t_{\text{FC}}{\left(F\right)}^{\alpha }{|F|}^{\beta }$$ (21)

for any α ≥ 1 and 0 ≤ β < 1/2.

In a similar mindset, we present an approximation preserving reduction of FC to the ILP problem in the subsequent section.

#### Approximation hardness of ILP

In this section, we show an approximation-preserving reduction of the FC problem to the problem of ILP. ILP is an NP-complete problem, in which many practically relevant combinatorial optimization tasks are formulated, such as planning or scheduling tasks (*33*). The problem is to minimize (or maximize) an objective function that depends on integer variables. In addition, there are constraints on the variables that need to be followed. Let us define an ILP problem within our formalism:

**Definition IV.10** [ILP problem (ILP)].

***Instance:*** An linear objective function over integer variables subject to linear constraints of the variables.

***Solution:*** A valid assignment of the variables under the constraints, such that the objective function is minimal.

We now show the reduction (τ_5_, *g*_2_) of FC to ILP by first giving the instance transformation τ_5_:

Let *F*(*z*_1_, …, *z_M_*) be a FC instance over variables *z*_1_, …, *z_M_* ∈ *ℕ*, which is a conjunction of *Q* clauses of the form (*z_u_* ≠ *z_v_*) and *R* clauses of the form [(*z_u_* = *z_v_*) → (*z_k_* = *z_l_*)] {which is equivalent to [(*z_u_* = *z_v_*) ∨ (*z_k_* ≠ *z_l_*)]}. For 1 ≤ *u*, *i* ≤ *M* and 1 ≤ *j* ≤ *R*, we introduce the ILP variables *w_i_*, *x*_*u*,*i*_, *a_j_*, *b_j_*, *s_j_* ∈ {0,1} and $1\leq {\hat{z}}_{u}\leq M$ , where ${\hat{z}}_{u}$ resembles the variable *z_u_* in *F*, *w_i_* indicates if the *i*th color is used, and *x*_*u*,*i*_ indicates if the variable ${\hat{z}}_{u}=i$ and *a_j_*, *b_j_*, *s_j_* are helper variables.

Note that for some *k*-coloring *P* = {*P*_1_, …, *P_k_*} of *F*, the clause (*z_u_* = *z_v_*) in *F* is true iff *z_u_*, *z_v_* ∈ *P_i_* for some color *i*. On the other hand, the clause (*z_u_* ≠ *z_v_*) in *F* is true iff *z_u_* ∈ *P_i_* and *z_v_* ∈ *P_i_* for some color *i*. In our ILP construction, we introduce an analog variable to *z_u_*, namely, ${\hat{z}}_{u}$ , where ${\hat{z}}_{u}$ directly takes as value the color *i*, i.e., ${\hat{z}}_{u}=i$ iff *z_u_* ∈ *P_i_*.

By our construction, we get the ILP problem ILP*_F_*

$$\text{minimize}\sum_{1\leq i\leq M}w_{i}$$ (22)

subject to the following constraints

$$\text{for all}\;u,i\in \left\{1,\ldots ,M\right\},\left(x_{u,i}=1\right)⟺\left({\hat{z}}_{u}=i\right)$$ (23)

$$\text{for all}\;u\in \left\{1,\ldots ,M\right\},\sum_{i=1}^{M}‍x_{u,i}=1$$ (24)

$$\text{for all}\;u,i\in \left\{1,\ldots ,M\right\},x_{u,i}\leq w_{i}$$ (25)

$$\text{for all}\;Q\;\text{clauses}\;\left(z_{u}\neq z_{v}\right)\;\text{and}\;\text{all}\;i\in \left\{1,\ldots ,M\right\},x_{u,i}+x_{v,i}\leq 1$$ (26)

$$\begin{array}{c} \text{for all}\;R\;\text{clauses}\;\left[\left(z_{u}\neq z_{v}\right)\vee \left(z_{k}=z_{l}\right)\right]\;\text{with}\;j\in \left\{1,\ldots ,R\right\}\\ \left(a_{j}=1\right)⟺\left({\hat{z}}_{k}={\hat{z}}_{l}\right) \end{array}$$ (27)

$$\left(b_{j}=1\right)⟺\left({\hat{z}}_{u}\neq {\hat{z}}_{v}\right)$$ (28)

$$s_{j}=\left(a_{j}\vee b_{j}\right)$$ (29)

$$s_{j}\geq 1$$ (30)

$$\text{and}\;w_{i},x_{u,i},a_{j},b_{j},s_{j}\in \left\{0,1\right\}\;\text{and}\;1\leq {\hat{z}}_{u},{\hat{z}}_{v},{\hat{z}}_{k},{\hat{z}}_{l}\leq M$$ (31)

Before explaining the constraints, let us note that for the sake of understanding, we display here logical clauses in Eqs. 23, 27, 28, and 29, although they are technically not ILP constraints. We refer to “Modelling logical clauses as inequality constraints” on how the logical clauses in Eqs. 23, 27, 28, and 29 are concretely converted to inequality constraints.

We define the binary variable *w_i_* to be 1 if color *i* is used. Hence, the minimization task at hand over the *w*_*i*′_*s* corresponds to finding the minimal coloring of *F*. Constraint Eq. 23 defines the binary variable *x*_*u*,*i*_ to be 1 iff ${\hat{z}}_{u}=i$ , i.e., indicating that *z_u_* ∈ *P_i_*. Constraint Eq. 24 ensures that any variable is assigned to exactly one color. Constraint Eq. 25 ensures that if there is some *z_u_* ∈ *P_i_*, then *w_i_* = 1 because color *i* is used. Constraint Eq. 26 encodes the (*z_u_* ≠ *z_v_*) clauses in *F*, i.e., that *z_u_*, *z_v_* are not assigned the same color. Constraints Eqs. 27, 28, 29, and 30 encode the [(*z_u_* ≠ *z_v_*) ∨ (*z_k_* = *z_l_*)] clauses in *F*.

In total, we get *M*(4*M* + *Q* + 1) + 12*R* constraints and 2*M*(*M* + 1) + 5*R* variables, which are polynomial in the size of *F*. Thus, τ_5_ is indeed computable in polynomial time. Now, the solution transformation *g*_2_ simply works by partitioning the variables *z_u_*, *z_v_* into the same set iff ${\hat{z}}_{u}={\hat{z}}_{v}$ . Clearly, *g*_2_ is computable in polynomial time. We show that (τ_5_, *g*) is indeed a reduction of FC to ILP by proving an even stronger result:

**Theorem IV.11** (Reduction of FC to ILP). Let τ_5_ be a polynomial-time algorithm that on input an instance F (*z*_1_, …. *z_M_*) of the FC problem FC outputs an instance ILPF of the ILP problem. Let *g*_2_ be a polynomial-time algorithm that on input an assignment A of ILPF outputs a coloring *P* of *F*. There exist τ_5_ , *g*_2_, such that *P* is a valid *k-*coloring of *F* if and only if *A* is a valid assignment of the variables in ILP*_F_*, such that the objective function of ILP*_F_* is *k.*

*Proof.* Let τ_5_ and *g*_2_ be the algorithms described in the beginning of this section.

⇒: We first prove that if *F* has a valid coloring *P* of *k* colors, then there exists an assignment *A* of the variables, such that

$$\sum_{1\leq i\leq M}w_{i}=k$$ (32)

Without the loss of generality, assume an ordering of the sets in ={*P*_1_, …. *P_k_*}. Because *P* is a coloring of *P*, the *Pi*’s are pairwise disjoint.

We assign the variables in ILPF as follows

$$\text{For all}\;i\in \left\{1,\ldots ,M\right\}\;w_{i}=1\left(i\leq k\right)$$ (33)

$$\text{for all}\;u\in \left\{1,\ldots ,M\right\}\;{\hat{z}}_{u}=\sum_{i=1}^{M}1\left(z_{u}\in P_{i}\right)\times i$$ (34)

$$\text{for all}\;u,i\in \left\{1,\ldots ,M\right\}\;x_{u,i}=1\left(z_{u}\in P_{i}\right)$$ (35)

$$\begin{array}{c} \text{for all}\;R,\text{clauses}\;[\left(z_{u}\neq z_{v}\right)V\left(z_{k}=z_{l})\right]\;\text{with}\;j\in \left\{1,\ldots ,R\right\},\\ a_{j}=1\left({\hat{z}}_{k}={\hat{z}}_{l}\right) \end{array}$$ (36)

$$b_{j}=1\left({\hat{z}}_{u}\neq {\hat{z}}_{v}\right)$$ (37)

$$s_{j}=a_{j}+b_{j}$$ (38)

Clearly, the objective function of ILP*_F_* is *k*. It remains to be shown that the constraints in ILPF are satisfied. First, note that from the variable assignments, it follows that $\left({\hat{z}}_{u}=i\right)⇐\Rightarrow \left[1\left(z_{u}\in P_{i}\right)=1\right]$ . We can then see that the constraint Eq. 23 is satisfied, because

$$\left(x_{u,i}=1\right)⇐\Rightarrow \left[1\left(z_{u}\in P_{i}\right)=1\right]⇐\Rightarrow \left({\hat{z}}_{u}=i\right)$$ (39)

The constraint Eq. 24 is satisfied due to the pairwise disjointedness of the sets in *P*, and we get

$$\sum_{i=1}^{M}x_{u,i}=\sum_{i=1}^{M}1\left(z_{u}\in P_{i}\right)=1$$ (40)

Next, we turn our attention to constraint Eq. 25. To see why this constraint is satisfied, observe the following: From the fact that $\Sigma_{i=1}^{M}x_{u,i}=1$ , it follows that there is exactly one *i*′, for which *x*_*u*,*i*′_ = 1. By the definition of *x*_*u*,*i*′_, we have 1 (*z_u_* ∈ *P*_*i*′_) = 1. Because *P* = {*P*_1_, …, *P_k_*} and *P*_*i*′_ is not empty, it must hold that *i*′ ≤ *k* and hence by construction *w*_*i*′_ = 1. For all other *i* ≠ *i*′, we have *x*_*u*,*i*_ = 0, and thus *x*_*u*,*i*_ ≤ *w_i_* and constraint Eq. 25 is satisfied. The constraint Eq. 26 is satisfied, since we have

$$x_{u,i}+x_{\mathrm{vi}}=1\left(z_{u}\in P_{i}\right)+1\left(z_{v}\in P_{i}\right)\leq 1$$ (41)

because of the assumption that *P* is a valid coloring, and this constraint occurs only for clauses of the form (*z_u_* ≠ *z_v_*). The constraint Eqs. 27 and 28 are satisfied by definition. One can easily see that constraint Eqs. 29 and 30 are also satisfied, since *P* is a valid coloring, and these constraints only occur for clauses of the form [(*z_u_* ≠ *z_v_*) ∨ (*z_k_* = *z_l_*)].

⇐: Assume that we are given a valid assignment *A* of the variables in ILP*_F_*, such that ∑_1≤*i*≤*M*_*w_i_* = *k*, then we can construct a valid coloring *P* for the corresponding FC instance *F*. To this end, *g*_2_ is run by partitioning the variables *z_u_*, *z_v_* into the same sets if ${\hat{z}}_{u}={\hat{z}}_{v}$ . Because

$$\sum_{1\leq i\leq M}w_{i}=k$$ (42)

there exist *i*_1_, …, *i_k_*, for which *w*_*i*1_, …, *w_ik_* = 1. Because for all *u* ∈{1, …, *M*}, we have

$$\sum_{i=1}^{M}x_{u,i}=1$$ (43)

and *x*_*u*,*i*_ ≤ *w_i_*, there exist *u*_1_, …, *u_k_*, for which $x_{u_{1},i_{1}}$, …, ≤ *x*_*u_k_*,*i_k_*_ = 1. Therefore, because the *u*_1_, …, *u_k_* are pairwise different and *i*_1_, …, *i_k_* are pairwise different, and because $\left(x_{u,i}=1\right)\Leftrightarrow \left({\hat{z}}_{u}=i\right)$ , there are ${\hat{z}}_{u_{1}}=i_{1},\ldots ,{\hat{z}}_{u_{k}}=i_{k}$ that are different from each other. Therefore, if we partition variables *z_u_*, *z_v_* into the same partition iff ${\hat{z}}_{u},{\hat{z}}_{v}$ , then we obtain exactly *k* partitions. Now, we need to show that this coloring is a valid coloring for *F*. The clauses (*z_u_* ≠ *z_v_*) are satisfied because constraint Eqs. 26 and 23 are satisfied. The clauses [(*z_u_* ≠ *z_v_*) ∨ (*z_k_* = *z_l_*)] are satisfied since constraint Eqs. 27, 28, 29, and 30 are satisfied. This ends the proof of the reduction.

Thus, we have that

$$\text{FC}\leq_{p}\text{ILP}$$ (44)

and in particular

$$\text{FC-RSA}\leq_{p}\text{ILP-RSA}$$ (45)

where ILP-RSA are the instances of ILP that we get when we apply τ_5_ to all instances of FC-RSA. Since by the same arguments as in “Approximation hardness of formula coloring” and since *g*_2_ transforms the minimal solution of ILP to the minimal solution of FC and ∣ILP*_F_*∣ = Θ(∣*F*∣^2^), the reduction (τ_5_, *g*_2_) preserves the approximation hardness of FC-RSA in the sense of the following theorem.

**Fig. 6.**
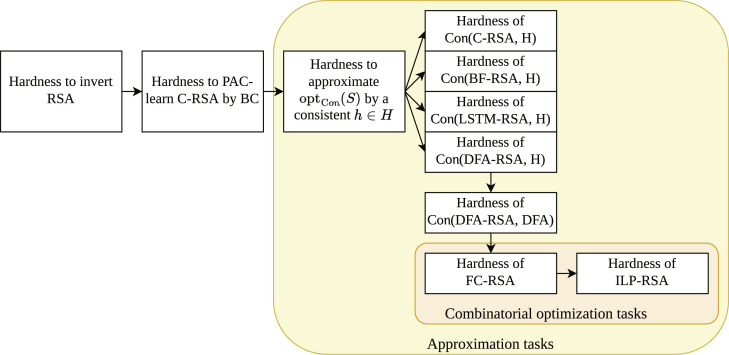
The argument chain that propagates the hardness to invert the RSA function to the hardness of approximating combinatorial optimization tasks.

**Theorem IV.12** (Classical hardness of approximation for ILP). Assuming the hardness of inverting the RSA function, there exists no classical probabilistic polynomial-time algorithm that on input an instance ILP*_F_* of ILP-RSA finds an assignment of the variables in ILP*_F_*, which satisfies all constraints and approximates the size opt_ILP_(ILP*_F_*) of the optimal solution by

$$\sum_{1\leq i\leq M}w_{i}\leq {\mathrm{opt}}_{\text{ILP}}{\left({\text{ILP}}_{F}\right)}^{\alpha }{|{\text{ILP}}_{F}|}^{\beta }$$ (46)

for any α ≥ 1 and 0 ≤ β < 1/4. To give a high-level overview of the hardness results established in this section, we present in Fig. 6 the chain of implications.

### Quantum efficiency

In the previous section, we have presented proofs for the classical hardness of various approximation tasks. In this section, we turn to showing a quantum advantage by proving that the instances resulting from the reductions described in “Classical hardness of approximation” can be solved in polynomial time given access to a fault-tolerant quantum computer. This yields the desired result of quantum separation for natural problems: Under the assumption that inverting the RSA function is hard, quantum computers can find close to optimal solutions to problem instances for which classical computers are incapable of findings solutions of the same quality.

First, we demonstrate that the solutions to instances of Con(C-RSA, BC) can be approximated by a polynomial factor in quantum polynomial time leveraging Shor’s algorithm. Later, we show approximation separation results for more “natural” problems, namely, FC and ILP.

**Theorem IV.13** [Quantum efficiency for approximating the solution of Con(C-RSA, BC)]. There exists a polynomial-time quantum algorithm that, on input of an instance *S* of Con(C-RSA, BC), finds a consistent hypothesis *h* ∈ BC, which approximates the size opt_Con_(*S*) of the optimal solution by

$$|h|\leq {\text{opt}}_{\text{Con}}{\left(S\right)}^{\alpha }$$ (47)

for all *S* and for some α ≥ 1.


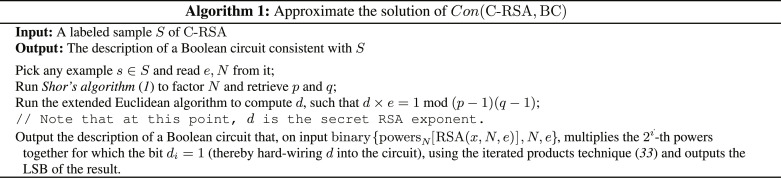


*Proof*. Let *S* be an instance of Con(C-RSA, BC). In Algorithm 1, on input *S*, a hypothesis circuit *h* is output, which is of size poly(*n*) and which explicitly decrypts a RSA ciphertext given by its power series. We know from “Approximation hardness of the Con problem” that *h* is consistent with *S* and of polynomial size. It is clearly the case that *n* ≤ opt_Con_(*S*), and thus it holds that

$$|h|\leq n^{\alpha }\leq {\text{opt}}_{\text{Con}}{\left(S\right)}^{\alpha }$$ (48)

Contrasted with the explicit approximation hardness from Theorem IV.6, this yields the super-polynomial advantage of quantum algorithms over classical algorithms for the specific approximation task, namely, approximating the optimal consistent hypothesis size by |*h*| with *h* consistent with *S*. We can indeed obtain similar results also for Con(BF-RSA, BF), Con(LSTM-RSA, LSTM), and Con(DFA-RSA, DFA). In particular, given *S*, we can use Algorithm 1 to obtain a consistent *h* of C-RSA and then leverage the poly-time instance transformations τ_1_, τ_2_, and τ_3_ to obtain an at most poly(*n*) larger approximation to the solution of Con(BF-RSA, BF), Con(LSTM-RSA, LSTM), and Con(DFA-RSA, DFA). Thus, we obtain the following corollary:

**Corollary IV.14** (Quantum efficiency for more approximation tasks). There exists a polynomial-time quantum algorithm that, on input an instance *S* of (i) Con(BF-RSA, BF), (ii) Con(LSTM-RSA, LSTM), or (iii) Con(DFA-RSA, DFA), finds a consistent hypothesis (i) *h* ∈ BF, (ii) *h* ∈ LSTM, and (iii) *h* ∈ DFA, which approximates the size optCon(*S*) of the optimal solution by

$$|h|\leq {\text{opt}}_{\text{Con}}{\left(S\right)}^{\alpha }$$

for all *S* and for some α ≥ 1*.*

This again yields super-polynomial advantages of quantum algorithms over classical algorithms for approximating the optimal solution size of the consistency problem by the size of a hypothesis that is consistent with the a sample. While this notion of approximation might seem unnatural, in the subsequent section, we turn our attention to approximating the solution of combinatorial optimization problems, for which it is natural to approximate some optimal scalar value while satisfying certain constraints.

#### Quantum advantage for combinatorial optimization

We now show a super-polynomial quantum advantage for approximating the solution of the combinatorial optimization task of FC. We have already established the classical approximation hardness of FC-RSA in Theorem IV.9 and give a polynomial-time quantum algorithm for approximating FC-RSA in the proof of the following theorem.

**Theorem IV.15** (Quantum efficiency for FC-RSA). There exists a polynomial-time quantum algorithm that on input of an instance FS of FC-RSA finds a valid coloring *P*, such that

$$|P|\leq {\text{opt}}_{\text{FC}}{\left(F_{S}\right)}^{\alpha }$$

*Proof*. Let us first describe how any instance *F_S_* of FC-RSA looks like. The overview of the construction of FC-RSA is that we started from class C-RSA of log-depth polysize Boolean circuits that explicitly decrypt an RSA ciphertext. The representation descriptions in C-RSA were then transformed using τ_1_, τ_2_, and τ_3_ to the class DFA-RSA. Thus, recall that any instance *S* of Con(DFA-RSA, DFA) is of the form

$$S=\left\{\left(\underset{=w_{i},\text{of length}\;p\left(n\right)\times \left(n^{2}+2n\right)\;\text{bits}}{\underbrace{\overset{p\left(n\right)}{\underset{l=1}{∥}}\text{binary}\left\{{\text{powers}}_{N}\left[\text{RSA}\left(x_{i},N,e\right)\right],N,e\right\},\underset{=b_{i}}{\underbrace{\text{LSB}\left(x_{i}\right)}}}}\right)|i=1,\ldots ,m\right\}$$ (49)

where || is the big concatenation of binary strings. Note that the repetition of *w_i_ p*(*n*) times comes from the reduction τ_3_, where for the construction of a DFA that simulates a log-space TM, the input needs to be repeated *p*(*n*) times.

Now, *F_S_* is obtained by the reduction (τ_4_, *g*_1_) from “Approximation hardness of formula coloring,” and *F_S_* is over the variables $z_{i}^{j},1\leq i\leq m,1\leq j\leq p\left(n\right)\times \left(n^{2}+2n\right)+1$ . Recall that $z_{i}^{j}$ encodes the state the DFA is in after reading bit *j* on input *w_i_*. By the construction of *F_S_*, we know that for each *i*_1_, *i*_2_ and *j*_1_, *j*_2_, such that

$$0\leq j_{1},j_{2}<p\left(n\right)\times \left(n^{2}+2n\right)+1$$ (50)

and $w_{i_{1}}^{j_{1}+1}=w_{i_{2}}^{j_{2}+1}$ , the following predicate

$$\left[\left(z_{i_{1}}^{j_{1}}=z_{i_{2}}^{j_{2}}\right)\to \left(z_{i_{1}}^{j_{1}+1}=z_{i_{2}}^{j_{2}+1}\right)\right]$$ (51)

occurs in *F_S_*. Note that *z*^0^ i is the starting state of the DFA.

Consider the bit $w_{1}^{n^{2}+n}$ , which is the LSB of *N*, for which we know that LSB(*N*) = $w_{1}^{n^{2}+n}=1$ , since *N* cannot be even. We know that for all other bits $w_{i_{2}}^{j_{2}+1}$ in *S* that are equal to $w_{1}^{n^{2}+n}$ , there occurs a predicate of the form

$$\left[\left(z_{1}^{n^{2}+n-1}=z_{i_{2}}^{j_{2}}\right)\to \left(z_{1}^{n^{2}+n}=z_{i_{2}}^{j_{2}+1}\right)\right]$$ (52)

in *F_S_*. Thus, by parsing *F_S_* and looking for all predicates of the form as in Eq. 52, we can infer all bits in *w_i_* given FS, for all *i*. Thus, we can reconstruct all *w_i_*’s from *F_S_*. Algorithm 2 does exactly this and runs in time poly(*n*) because there are *O*(|*S*|^2^) clauses in *F_S_*.


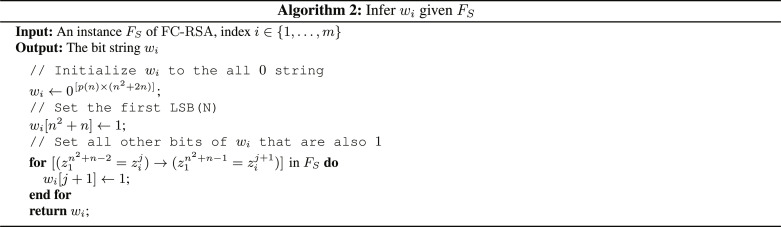


Remember that our goal in this proof is to give a polynomial-time quantum algorithm that on input an *F_S_* finds a valid coloring of size less than opt_FC_(*F_S_*)^α^. At this point, we have described how the instances *F_S_* look like and how we can extract the *w_i_*’s from it. After having obtained a *w_i_* from *F_S_*, we read *e* and *N* from it and then construct the Boolean circuit *c* by the same technique used in Algorithm 1. Note that *c* is exactly of the form of Boolean circuits in C-RSA, from which we originally constructed FC-RSA. When presented the input binary {powers*N* [RSA(*x_i_*, *N*, *e*)], *N*, *e*}, *c* outputs LSB(*x_i_*). We can transform *c* into a DFA that is consistent with *S* and then find a coloring for *F_S_* from that DFA. Therefore, to obtain a DFA that is consistent with *S*, we run *c* through the instance transformations *t*′ = τ_3_{τ_2_[τ_1_(c)]} to obtain the DFA *t*′, which is consistent with *S* and of size poly(*n*). On input *w_i_*, *t*′ accepts if LSB(*x_i_*) = 1 and rejects if LSB(*x_i_*) = 0. Now, we minimize *t*′ using the standard DFA minimization algorithm (*26*) to obtain the smallest and unique DFA *t*, which accepts the same language as *t*′ and thus is also consistent with *S* and of minimal size. This DFA minimization is in principle not needed for the proof, but it is a further optimization step.

We then run Algorithm 3 to obtain a coloring for *F_S_* from *t*. The DFA *t* consists of the set of states *Q*, the set of input symbols Σ = {0, 1}, the set of accepting states ω ⊆ *Q*, the start state *q*_0_ ∈ Q, and the transition function λ that takes as arguments a state and an input symbol and returns a state (*26*). Furthermore, without loss of generality, we fix an ordering of the states in *Q* = 0, …, *k* − 1 with *q*_0_ = 0. We can convince ourselves that the result of Algorithm 3 is indeed a valid coloring for *F_S_* because it assigns $z_{i_{1}}^{j_{1}}$ and $z_{i_{2}}^{j_{2}}$ the same color if and only if t is in the same state after reading $w_{i_{1}}^{j_{1}}$ on input *w*_*i*1_ and after reading $w_{i_{2}}^{j_{1}}$ on input *w*_*i*2_. Therefore, a conjunct

$$\left[\left(z_{i_{1}}^{j_{1}}=z_{i_{2}}^{j_{2}}\right),\to ,\left(z_{i_{1}}^{j_{1}+1}=z_{i_{2}}^{j_{2}+1}\right)\right]$$ (53)

cannot be violated since it appears in *F_S_* only if $w_{i_{1}}^{j_{1}+1}=w_{i_{2}}^{j_{2}+1}$ and, by Algorithm 3, if $z_{i_{1}}^{j_{1}}$ is assigned the same color as $z_{i_{2}}^{j_{2}}$ , then $z_{i_{1}}^{j_{1}+1}$ and $z_{i_{2}}^{j_{2}+1}$ have the same color (*21*). In addition, a conjunct

$$\left[z_{i_{1}}^{p\left(n\right)\times \left(n^{2}+2n\right)},\neq ,z_{i_{2}}^{p\left(n\right)\times \left(n^{2}+2n\right)}\right]$$ (54)

cannot be violated because it appears only if $b_{i_{1}}\neq b_{i_{2}}$ and if $z_{i_{1}}^{p\left(n\right)\times \left(n^{2}+2n\right)}$ would be assigned the same color as $z_{i_{2}}^{p\left(n\right)\times \left(n^{2}+2n\right)}$ , then t would be in the same state after reading all bits of *w*_*i*_1__ and *w*_*i*_2__, which is either an accepting or rejecting state, which in turn contradicts that t is consistent with *S* and *b*_*i*_1__ ≠ *b*_*i*_2__ (*21*). It follows that the coloring obtained through Algorithm 3 is upper bounded by opt_FC_(*F_S_*)^α^ for some α because *t* has polynomial size with the number of states given by *k* = ∣*Q*∣ ≤ *n*^α^ ≤ opt_Con_(*S*)^α^ = opt_FC_(*F_s_*)^α^ and Theorem IV.8.


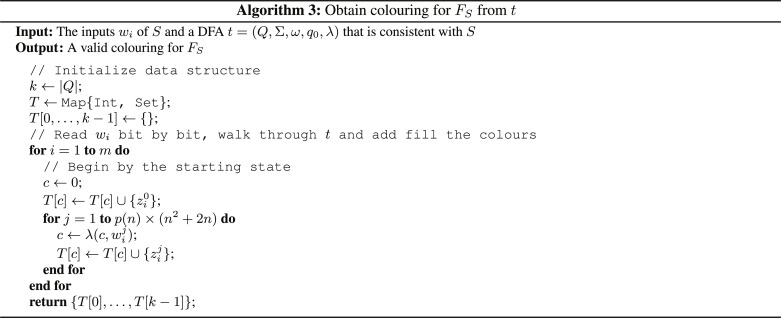


Thus, because of Theorem IV.9 and IV.15, we have the super-polynomial quantum advantage for approximating a combinatorial optimization solution.

Note that whether an instance *I* of FC belongs to the set FC-RSA can be decided in quantum polynomial time. To see why, for a given FC instance *I*, it can be decided in quantum polynomial-time whether the instance is also contained in FC-RSA, consider the following algorithm A. First, A tries to reconstruct the RSA parameters *N*,*e*, and the ciphertext-label pairs from *I*. If these parameters cannot be reconstructed from *I* (because it does not follow the correct structure), clearly *I* ∉ FC-RSA. If *A* can reconstruct the respective parameters, then *A* constructs a Con(DFA, DFA) instance and then applies the described reduction chain to create an instance of FC-RSA. If the resulting instance matches instance *I*, clearly *I* ∈ FC-RSA and can therefore be solved by algorithm 3. We reuse the techniques used above to prove the super-polynomial quantum advantage for approximating the optimal solution of an ILP problem, namely, ILP*_F_S__*∈ ILP-RSA.

**Theorem IV.16** (Quantum efficiency for ILP-RSA). There exists a polynomial-time quantum algorithm that, on input an instance ILP*_F_S__*of ILP-RSA, finds a variable assignment *A* that satisfies all constraints and for which the objective function is bounded as

$$\sum_{1\leq i\leq M}w_{i}\leq {\text{opt}}_{\text{ILP}}{\left({\text{ILP}}_{F}\right)}^{\alpha }$$

for all ILP*_F_S__* and for some α ≥ 1.

*Proof*. Given an instance ILP*_F_S__*, one can easily reconstruct FS from the constraint Eq. 26 to 30 in polynomial time. It is then possible to obtain a valid coloring *P* of *F_S_*, given the routine described in the proof for Theorem IV.15, such that |*P*| ≤ opt_FC_(*F_S_*). With *P*, we can get a valid assignment of the variables in ILP*_F_S__*using the routine described in the ⟹-direction in the proof of Theorem IV.11. Also, due to Theorem IV.11, we know that this variable assignment admits the objective function of ILP*_F_S__*to be less than *opt*_ILP_(ILP*_F_S__*)^α^ = opt_FC_(*F_S_*)^α^.

Thus, because of the classical approximation hardness from Theorem IV.12, we encounter a super-polynomial quantum advantage for approximating the solution of an ILP problem. It is important to stress that the reduction is explicit: That is to say, we can construct the instances for which one can achieve a quantum advantage of this kind.

### The optimization problem in terms of a quantum Hamiltonian

The quantum algorithm presented is distinctly not of a variational type, as they are commonly proposed for approximating combinatorial optimization tasks using a quantum computer (*10*). That said, it is still meaningful to formulate the problem at hand as an energy minimization problem, to closely connect the findings established here to the performance of variational quantum algorithms (*11*, *12*) in near-term quantum computing, as this is the context in which these problems are typically stated. It remains to be investigated to which extent the resulting instances can be practically studied and solved on near-term quantum computers. The aim here is to provide a formal connection from FC and ILP problems to variational quantum algorithms, where the problems are commonly stated as unconstrained binary optimization problems of the form

$$x^{*}≔{\text{argmin}}_{x\in {\left\{0,1\right\}}^{n}}f\left(x\right)$$ (55)

where *f* : {0,1}*^n^* → ℝ is an appropriate cost function and *x*^∗^ is a solution bit string of *f*. Particularly common are quadratic unconstrained binary optimization problems

$$\text{minimize}\;f\left(x\right)=x^{T}\mathrm{Qx}$$ (56)

$$x^{*}≔{\text{argmin}}_{x\in {\left\{0,1\right\}}^{n}}f\left(x\right)$$ (57)

where *Q* = *Q^T^* is a real symmetric matrix. It is a well-known result that all higher order polynomial binary optimization problems can be cast into the form of such a quadratic unconstrained binary optimization problem, possibly by adding further auxiliary variables; However, it can also be helpful to keep the higher order polynomials. All these problems can be directly mapped to Hamiltonian problems. Notably, for quadratic unconstrained binary optimization problems, the minimum is equivalent with the ground state energy of the quantum Ising Hamiltonian defined on *n* qubits as

$$H=\sum_{i,j=1}^{n}Q_{i,j}\left(1-Z_{i}\right)\left(1-Z_{j}\right)$$ (58)

where *Z_j_* is the Pauli-*Z* operator supported on site labeled *j*. For higher order polynomial problems, one can proceed accordingly.

Let us, pars pro toto, show how the FC problem in the center of this work can be cast into a quartic binary optimization problem. Let *k* ∈ ℕ be an upper bound to the number of colors used for a formula over m variables, with *z*_1_, …, *z_m_* ∈ {1, …, *k*} being the variables in the formula. We can then make use of *n* = *mk* bits (which then turn into *n* = *mk* qubits). These bits referred to as *b_v,c_* feature the double labels (*v*, *c*), where *v* ∈ {1,...,*m*} labels the vertices and *c* ∈ {1,...,*k*} the colors. If the vertex *v* is assigned the color *c*, we set *b*_*v*,*c*_ = 1, and *b_v,d_* = 0 for all *d* ≠ *c*. To make sure that the solution will satisfy such an encoding requirement, one adds a penalty of the form ${\left(1-\sum_{c=1}^{k}b_{v,c}\right)}^{2}$ . The clauses of the form (*Z_i_* ≠ *Z_j_*) are actually precisely like in the graph coloring problem (*34*). This can be incorporated by penalty terms of the type $\sum_{c=1}^{k}b_{z_{i},c}b_{z_{j},c}$ : Then, equal colors are penalized by energetic terms. The second type of clause requires more thought: Exactly if (*Z_i_* = *Z_j_*) is true and (*Z_j_* = *Z_k_*) is false, there should be a Hamiltonian penalty. Hence, this is a quadratic Boolean constraint of the form

$$\left(\sum_{c=1}^{k}b_{z_{i},c}b_{z_{j},c}\right)\left(1-\sum_{d=1}^{k}b_{z_{i},d}b_{z_{k},d}\right)$$ (59)

Again, this can be straightforwardly be incorporated into a commuting classical Hamiltonian involving only terms of the type (1 − *Z_j_*) for suitable site labels *j*, precisely as commonly considered in quantum approximate optimization (*10*). Last, to ensure we find a minimal coloring, we can either run the quantum optimization algorithm for increasing *k* and check whether a valid coloring has been found or one adds additional *k* qubits *w_c_*, *c* ∈ {1, …, *k*}, which we enforce to be 1 if color *c* is used and 0 if color *c* is not used by adding the energetic penalty *b*_*v*, *c*_ − *b*_*v*, *c*_*w_c_* for all *v* ∈ {1, …, *m*}, *c* ∈ {1, …, *k*}. This corresponds to enforcing the inequality *b*_*v*, *c*_ ≤ *w_c_*. We can then add the energetic penalty $\sum_{c=1}^{k}w_{c}$ to enforce the optimization algorithm to find the minimal coloring. For these reasons, the approximation results proven here motivate the application of quantum optimization techniques for commuting Hamiltonian optimization problems. Note that this construction is very similar to the integer linear program we proposed in “Approximation hardness of integer linear programming” to reduce the FC problem to ILP.

Because any combinatorial optimization problem of the type discussed here can be mapped to a local Hamiltonian, it is apparent that the local Hamiltonian problem is NP-hard. It is even known to be QMA-complete (*35*), which is at least as hard as NP. However, for the FC-RSA instances—which give rise to a specific subclass of local Hamiltonians—it remains to be studied how well the corresponding Hamiltonians can be solved using quantum optimization algorithms in practice.

### Modeling logical clauses as inequality constraints

In this section, we present some details of proofs that are made reference to in the main text. To model the logical Boolean operator ∨, such that *s*: = (*a* ∨ *b*) for binary variables *s*, *a*, and *b*, we require the inequality constraints

s≥a (60)

s≥b (61)

s≤a+b (62)

which is easily seen as being equivalent.

We are here interested in modeling logical equivalences of the form $\left(a=1\right)\Leftrightarrow \left({\hat{z}}_{u}={\hat{z}}_{v}\right)$ and $\left(b=1\right)\Leftrightarrow \left({\hat{z}}_{u}\neq {\hat{z}}_{v}\right)$ for the binary variables *a*, *b* and integers ${\hat{z}}_{u}={\hat{z}}_{v}$ . For the former, we model the forward and backward implications as follows.

$\left(a=1\right)\Rightarrow \left({\hat{z}}_{u}={\hat{z}}_{v}\right)$ : Choose a large enough constant *L*, such that ${\hat{z}}_{u}={\hat{z}}_{v}\leq L$ , and then, because ${\hat{z}}_{u}={\hat{z}}_{v}\geq 0$ , the following constraints encode the implication

$${\hat{z}}_{u}\leq {\hat{z}}_{v}+\left(1-a\right)L$$ (63)

$${\hat{z}}_{u}\geq {\hat{z}}_{v}-\left(1-a\right)L$$ (64)

Clearly, the constraint Eqs. 63 and 64 are satisfied for ${\hat{z}}_{u}={\hat{z}}_{v}$ if *a* = 1 and for any ${\hat{z}}_{u}={\hat{z}}_{v}$ if *a* = 0. $\left({\hat{z}}_{u}={\hat{z}}_{v}\right)\Rightarrow \left(a=1\right)$ : Note that this implication is equivalent to $\left(a\neq 1\right)\Rightarrow \left({\hat{z}}_{u}\neq {\hat{z}}_{v}\right)$ , which again is equivalent to $\left(a\neq 1\right)\Rightarrow \left[\left({\hat{z}}_{u}>{\hat{z}}_{v}\right),V,\left({\hat{z}}_{u}<{\hat{z}}_{v}\right)\right]$ , which we will model below. We introduce a new binary variable *q*, for which if *a* = 0 and *q* = 1 then ${\hat{z}}_{u}<{\hat{z}}_{v}$ , and if *a* = 0 and *q* = 0, then ${\hat{z}}_{u}>{\hat{z}}_{v}$ . This can be modeled by the constraints

$${\hat{z}}_{u}<{\hat{z}}_{v}+\left(1-q+a\right)L$$ (65)

$${\hat{z}}_{u}>{\hat{z}}_{v}+\left(q+a\right)L$$ (66)

The constraint Eqs. 65 and 66 are satisfied for ${\hat{z}}_{u}\neq {\hat{z}}_{v}$ if *a* = 0 and for any ${\hat{z}}_{u},{\hat{z}}_{v}$ if *a* = 1. The variable *q* essentially indicates if ${\hat{z}}_{u}<{\hat{z}}_{v}$ or if ${\hat{z}}_{u}>{\hat{z}}_{v}$ when *a* = 0 and can be ignored after the optimization process. In a similar manner to the constraints above, we can model $\left(b=1\right)\Leftrightarrow \left({\hat{z}}_{u}\neq {\hat{z}}_{v}\right)$ as

$${\hat{z}}_{u}<{\hat{z}}_{v}+\left(2-q\prime -b\right)L$$ (67)

$${\hat{z}}_{u}>{\hat{z}}_{v}+\left(1+q\prime -b\right)L$$ (68)

$${\hat{z}}_{u}\leq {\hat{z}}_{v}+\mathrm{bL}$$ (69)

$${\hat{z}}_{u}\geq {\hat{z}}_{v}-\mathrm{bL}$$ (70)

in terms of inequality constraints.
